# Knowledge, attitudes and practices of UK paramedics regarding pharmacology and the legal, management and administration aspects of medicines: a cross-sectional online quantitative survey

**DOI:** 10.29045/14784726.2020.09.5.2.1

**Published:** 2020-09-01

**Authors:** Samantha Laws, Chao Wang, Mary Halter

**Affiliations:** Kingston University and St George’s University of London ORCID iD: https://orcid.org/0000-0002-0570-2015; Kingston University and St George’s University of London ORCID iD: https://orcid.org/0000-0002-7873-0594; Kingston University and St George’s University of London ORCID iD: https://orcid.org/0000-0001-6636-0621

**Keywords:** drug administration routes, emergency medical technicians, pharmacology

## Abstract

**Introduction:**

Changes in the paramedic profession have seen an increased range of medicines available within UK ambulances services. However, poor practice in medicines management has been identified by the Care Quality Commission. Literature in this area is sparse. This study aimed to determine the perceived knowledge, attitudes and practices of paramedics regarding pharmacology and the legal and regulatory issues of medicines management and administration.

**Methods:**

The study utilised a cross-sectional survey design, administering an anonymous online survey to all (approximately 1000) paramedics within one UK NHS ambulance trust. The survey focused on paramedic knowledge on pharmacology, legal supply and administration; self-assessment of knowledge and confidence related to medicine management and administration; and personal characteristics. The primary outcome was percentage of (pre-determined) correct answers.

**Results:**

251 responses were received. The mean percentage of correct answers was 79.0% (SD 10.0), with variation by question observed, from 34.7 to 97.2% correct responses. A higher correct knowledge was associated with: higher self-rated confidence, lower self-reported knowledge, being less likely to report errors and higher education-based initial route into the paramedic profession.

**Conclusion:**

This single-site UK-based survey highlighted variation in medicines knowledge among self-selecting paramedic respondents. The results indicate a need for medicines-specific further education for all paramedics, particularly those who have not experienced longer formal education entry routes, integrating a focus on confidence and self-perceived knowledge, and enhancing and embedding integrated improvement strategies. Further research is required with larger, multi-site samples, and to evaluate the impact of education packages developed.

## Introduction

Internationally, the paramedic profession is undergoing professionalisation that has seen a transformation from ambulance drivers to highly skilled and knowledgeable healthcare professionals within a 20-year period (Duffy & Jones, 2017). Professionalisation of the paramedic is recognised to bring rewards such as remuneration, respect and knowledge, and to carry increased accountability and responsibility, dependent upon higher education and clinical leadership (First et al., 2013). Alongside this, the ambulance service has increasingly been recognised as contributing to an integrated urgent and emergency care health system, including achieving efficiencies through the reduction of clinically unnecessary patient visits to the Emergency Department (ED) (Department of Health, 2017). Within the integrated system, paramedics are seeing and treating more patients independently, either to prevent conveyance of the patient or to initiate treatment prior to arrival at the ED, taking on a greater breadth of clinical work yielding a wider range of treatment options (NHS England, 2015b).

Medicines have been a part of this transformation. Paramedics first used medicines by following a rigid protocol-based system, and this progressed to using medicines by exemptions according to schedules 17 and 19 of the Human Medicines Regulations 2012 (HM Government, 2012). Now there are many more medicines available under patient group directions (PGDs) (England, 2016), which allow a greater flexibility for individual ambulance trusts to tailor their use of medicines to their patients’ needs and to allow certain specialist paramedics to administer additional medicines required for their roles. In addition, independent prescribing is now available for certain paramedics. The UK College of Paramedics recognises the necessity of the paramedic profession to continue to promote excellent, underpinning knowledge and professional standards to optimise safe care for patients (College of Paramedics, 2017), and to support independent prescribing (NHS England 2013, 2015a).

NICE (2015) notes that optimisation of medicines use is dependent upon safety, with a high human and financial cost associated with errors, and a number of causes for those errors, listing ‘lack of knowledge, failure to follow systems and protocols, interruptions (for example, during prescribing, administration and dispensing), staff competency, poor instruction, and poor communication’. Poor practice in medicines management in UK NHS ambulance services, and a requirement to embed safety procedures pertaining to medicines into everyday practice that can be sustained by front line ambulance staff and managers, have been identified by the Care Quality Commission (CQC, 2017a, 2017b).

Focusing just on lack of knowledge as one of the known causes for medicines safety issues, the literature on paramedic pharmacology knowledge and issues related to medicines management is sparse; however, three studies suggest that there are areas warranting concern internationally. An Australian study of 20 intensive care course paramedics demonstrated a potential risk to patient safety in calculating drug doses (Boyle & Eastwood, 2018); a study of 142 paramedics on simulated paediatric patients with anaphylaxis uncovered multiple causes of medication errors issues (Lammers et al., 2014); and another US study using an anonymous online survey with 352 respondents reported that 9.1% had made medication errors in the past 12 months, with 4% never having been reported (Vilke et al., 2007). No literature has been located that examines the knowledge paramedics have surrounding medicines, how conversant they are with the procedures and policies in place surrounding medicines or what stage of implementation such procedures have reached. It is also not known whether these factors vary according to paramedic education route or experience.

This study aims to determine the perceived knowledge, attitudes and practices of paramedics regarding pharmacology and the legal and regulatory issues of medicines management and administration within UK NHS ambulance trusts, and if that knowledge is associated with length of service and educational route to becoming a paramedic.

## Methods

### Study design, population and selection of participants

The study utilised a cross-sectional survey design conducted using an anonymous online self-report survey hosted by Online Surveys.

The survey was designed by the research team in collaboration with members of ambulance trust medicines management groups, trust education team paramedics and paramedic education staff, with input from patient and public involvement representatives of the ambulance trust and the research team’s university. It was tested with five university-based paramedic lecturers for face validity.

The study population comprised all approximately 1000 paramedics within one UK NHS ambulance trust. The trust covers a geographical area including densely populated urban areas and sparsely populated rural areas, and responds to over three quarters of a million calls per year. In the absence of published data upon which to base assumptions of knowledge level or any intra-profession differences, a formal sample size calculation could not be performed.

An invitation to participate in the survey, including the URL, was advertised by the ambulance trust via its online closed group platforms for clinical staff and via posters placed in every ambulance station, headquarters, emergency operations centre and 111 call centre. The invitation stated that the survey would take approximately 20 minutes to complete, and included the researcher’s details if more information was required or to obtain the Participant Information Sheet (PIS). The invitation wording is shown in Appendix 1. A paper version of the survey was offered in the invitation. A £25 voucher prize draw was offered as an incentive to participate. The online survey was open from 28 January to 20 March 2019.

Favourable ethical opinion was gained from the Faculty Research Ethics Committee of the joint Faculty of Health, Social Care and Education at Kingston University and St George’s, University of London (ref number 2018-11-003). Research governance approval was achieved from the Health Research Authority, with Capability and Capacity agreed by the ambulance trust. The study was registered on the NIHR portfolio.

### Survey technical details

The survey comprised four sections:

- The first included the introduction, the PIS and a consent page, directing respondents to a page explaining their ineligibility for the survey if they answered no to a consent question.
- The second (main section) focused on paramedic knowledge, using a set of questions presenting scenarios encompassing the pharmacology of medicines and the legal supply and administration thereof, in which the paramedic marked what they considered to be the best answer. A short Likert-style section required respondents to answer questions on how they felt about their abilities in pharmacology and the legal and regulatory issues related to medicine management and administration. These questions were allocated to ‘domains’ of knowledge and attitude.
- The third collected details about the paramedic’s personal characteristics: educational route to becoming a paramedic, length of service, age and gender.
- The last section contained a link to a separate survey for the prize draw, whereby the names and email addresses of the prize draw respondents could not be linked to the anonymous medicines survey responses.

All main section survey questions had an ‘I don’t know’ option, and personal characteristic questions had a ‘prefer not to answer’ option. Respondents could navigate around the survey, including going back to change previous answers.

Survey responses were downloaded as raw individual entries from Online Surveys as an Excel file, and statistical analyses were conducted in Stata 14.2. Recruitment rate was calculated using the number of surveys in which the first question after the consent page was opened divided by the number of clicks on the first page. Completion rate was calculated using the number of surveys in which the last page was submitted divided by the number of surveys in which the first question after the consent page was opened. Only completed surveys were available for analysis due to conditions imposed to comply with data regulations. To comply with data regulations, no cookies were used and no IP address check was conducted; therefore, no log file analysis was conducted for identification of multiple entries. No timeframe cut-off point was employed.

### Statistical analysis

Each knowledge question had a pre-determined ‘correct’ answer, and responses to each question were categorised as ‘correct’ or ‘incorrect’ against this proforma. ‘I don’t know’ was coded as incorrect. A total score of 16 points was possible. This score formed the main outcome for the study: the percentage of correct answers.

Attitude scores related to perceived knowledge, confidence and the perception of need for more teaching were combined separately, to form one rating for the overall attitude for each area.

Questions not answered were treated as missing data. For length of service, the data were converted into months, but where the answers were not clear, data were marked as missing.

Personal characteristic variables, such as age and gender, and attitude variables were firstly reported using descriptive statistics. The impact of personal characteristic and attitude variables on the outcome variable was investigated using linear regression models. The dependent variable (percentage of correct answers) was transformed using the logit function to improve normality and reflect the bounded nature (between 0 to 1) of such variable (Papke & Wooldridge, 1996). A series of common linear model diagnostics were performed, including normality of residuals, collinearity (using variance inflation factors), heteroscedasticity and omitted variable bias. Additionally, to explore the impact of missing data, Little’s chi-squared test was used to test whether the missingness is completely at random (MCAR). In the presence of outliers, after inspecting the leverage-versus-squared-residual plot, a robust regression was fitted. Because of the transformation of the outcome variable, the interpretation of the modelling results is not straightforward. To facilitate interpretations, the marginal effect (i.e. derivative) of the continuous independent variable on the dependent variable and their 95% confidence intervals (CIs) were calculated. Likewise, for the categorical independent variable, discrete change from the reference category on the outcome variable was calculated. To account for the multiple testing problem, the false discovery rate (FDR) was also calculated using the Benjamini–Hochberg method, to measure how likely a statistically significant finding (p < 0.05 in this article) was false.

## Results

### Respondents/demographics

**Figure 1:**
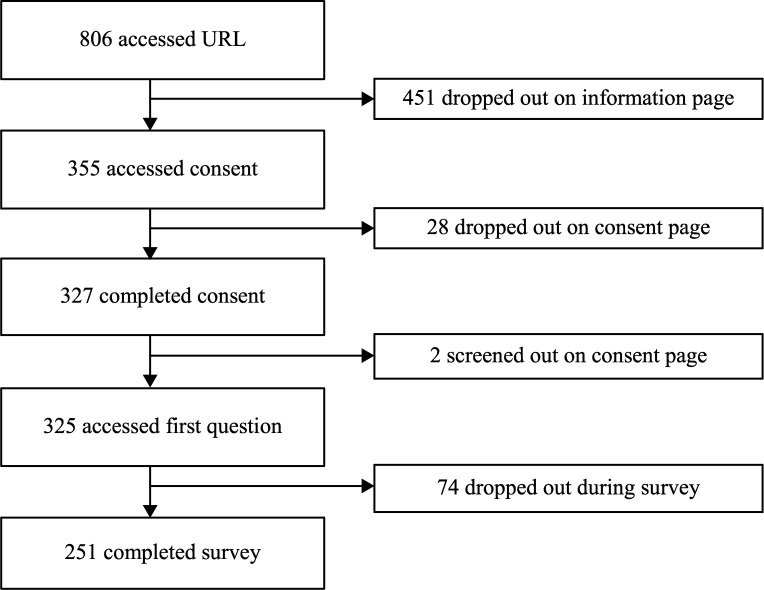
Figure 1. Flow chart of respondent progress, detailing drop-out points.

The survey URL was accessed 806 times. Two respondents were screened out on the consent page and a further 477 did not proceed beyond accessing the first page (consent). 251 responses to the electronic survey were received (Figure 1). No paper surveys were requested. The recruitment rate was 40.3% (325/806), and the completion rate 77.2% (251/325).

Respondent characteristics are shown in Table 1.

**Table 1.** Respondent characteristics.

	Total
Respondent characteristic and descriptive statistic	(N = 251)
**Age (years)**	
Mean (SD)	36.15 (9.31)
Median (Q1, Q3)	34.0 (29.0, 43.0)
Min, Max	22.0, 60.0
Missing	6
	
**Length of service (years)**	
Mean (SD)	9.59 (7.97)
Median (Q1, Q3)	7.7 (3.1, 14.0)
Min, Max	0.3, 35.3
Missing	35
	
**Gender**	
Male	132 (52.6%)
Female	98 (39.0%)
Prefer not to say	14 (5.6%)
Missing	7 (2.8%)
	
**Route to become a paramedic**	
In-service training	60 (23.9%)
Diploma of Higher Education	7 (2.8%)
Foundation degree in Paramedic Science	89 (35.5%)
BSc or BSc (Hons) degree in Paramedicine	87 (34.7%)
Missing	8 (3.2%)

SD: standard deviation; Q1: 25th percentile; Q3: 75th percentile.

## Knowledge of pharmacology and medicines management

Of 251 responses, 243 contained answers to knowledge questions. The mean percentage of correct answers was 79.0% (Table 2).

**Table 2.** Total knowledge score.

Percentage of correct answers	
Mean (SD)	79.0 (10.0)
Median (Q1, Q3)	81.0 (72, 88)
Min, Max	52, 100
N	243

SD: standard deviation; Q1: 25th percentile; Q3: 75th percentile; N: total non-missing observations.

Within the overall correct response percentage, variation by question was observed, from a low of 34.7% for the ‘action when finding a medicine in an ambulance cupboard that should not be there’ to a high of 97.2% for ‘reason for administration of diazepam and morphine with extreme caution to the same patient on the same occasion’ and ‘proficiency in the route of administration’ in an end-of-life care patient. Table 3 shows the percentage of correct answer scores to individual questions and points allocation.

**Table 3.** Correct answer scores to individual questions.

Topic of question	Score for that question/points	Incorrect answers n (%)	Correct answers n (%)	Missing n (%)
Process name for a drug passing across the gastrointestinal tract	1	28 (11.2%)	218 (86.9%)	5 (2.0%)
Part of the body in which most drugs are metabolised	1	18 (7.2%)	228 (90.8%)	5 (2.0%)
Why glyceryl trinitrate (GTN) is never given as a tablet to swallow	1	71 (28.3%)	175 (69.7%)	5 (2.0%)
Meaning of the term ‘half-life’	1	33 (13.1%)	212 (84.5%)	6 (2.4%)
Reason for administration of diazepam and morphine with extreme caution to the same patient on the same occasion	1	2 (0.8%)	244 (97.2%)	5 (2.0%)
When to call out a Critical Care Paramedic for ketamine when attending a patient with an isolated limb fracture	1	93 (37.1%)	158 (62.9%)	0 (0%)
Action when finding a medicine in an ambulance cupboard that should not be there	1	159 (63.3%)	87 (34.7%)	5 (2.0%)
**Delegating tasks**				
Drawing up of morphine delegated to a crew mate who is not a registered paramedic	0.2	14 (5.6%)	237 (94.4%)	0 (0%)
Mixing of hydrocortisone powder with water for IM injection delegated to a crew mate who is not a registered paramedic	0.2	57 (22.7%)	194 (77.3%)	0 (0%)
Injection of benzylpenicillin delegated to a student paramedic	0.2	60 (23.9%)	191 (76.1%)	0 (0%)
Transfer of a patient to hospital to whom you administered diazepam delegated to a crew mate who is not a registered paramedic	0.2	43 (17.1%)	208 (82.9%)	0 (0%)
Allowing the use of your tranexamic acid to treat the patient of a GP who is suffering from a serious gastrointestinal bleed delegated to the GP	0.2	59 (23.5%)	192 (76.5%)	0 (0%)
Action for a patient who is not on the inclusion list for a medicine for which there is a PGD	1	64 (25.5%)	187 (74.5%)	0 (0%)
**Actions needed when attending a patient with an end of life care package with a prescription and administration**				
Proficiency in the route of administration	0.25	7 (2.8%)	244 (97.2%)	0 (0%)
Contact the prescriber	0.25	41 (16.3%)	210 (83.7%)	0 (0%)
Complete the chart	0.25	11 (4.4%)	240 (95.6%)	0 (0%)
Ensure knowledgeability and confidence about the medicine before giving it	0.25	14 (5.6%)	237 (94.4%)	0 (0%)
Actions on arriving home with morphine	1	90 (35.9%)	161 (64.1%)	0 (0%)
Leaving medications to take later	1	14 (5.6%)	237 (94.4%)	0 (0%)
LEGAL limit of morphine sulphate to one patient	1	65 (25.9%)	186 (74.1%)	0 (0%)
**Legal frameworks, guidelines or evidence-based tools to optimise the use of medicines**				
JRCALC guidelines	0.25	20 (8.0%)	231 (92.0%)	0 (0%)
Trust PGDs	0.25	23 (9.2%)	228 (90.8%)	0 (0%)
NICE guidelines	0.25	98 (39.0%)	153 (61.0%)	0 (0%)
British National Formulary (BNF)	0.25	70 (27.9%)	181 (72.1%)	0 (0%)
Reporting of medication-related adverse events	1	116 (46.2%)	135 (53.8%)	0 (0%)
Correct INITIAL route of administration of adrenaline in patients with anaphylaxis	1	3 (1.2%)	242 (96.4%)	6 (2.4%)

## Association of knowledge score and attitude with respondent characteristics

We found no violations of linear model assumptions and no evidence that there was a violation of MCAR assumption (Little’s chi-squared test p = 0.25). A low FDR (around 0.03) was found when a p-value less than 0.05 was considered statistically significant.

Table 4 shows the marginal effect (or discrete change for categorical variables) of various predictors on the percentage of correct answers, indicating how much the knowledge score changed given a unit change in personal characteristics and attitudes of respondents.

**Table 4.** Marginal effects of variables on knowledge score in percentage points.

	Marginal effects	95% confidence interval	*p*-value
*Route to paramedic*			
BSc or BSc (Hons)	5.51*	1.04 to 9.98	0.016
Foundation degree	4.38*	0.76 to 8.00	0.018
Other routes	*Reference*		
			
Length of service (years)	-0.19	-0.49 to 0.11	0.222
Total perceived confidence score	1.08***	0.47 to 1.69	< 0.001
Total perceived knowledge score	-0.74*	-1.35 to -0.13	0.018
Total perceived need for teaching score	-0.90***	-1.42 to -0.38	0.001
			
*How likely to report giving the wrong medicine with no harm caused to the patient*			
Somewhat likely	-1.32	-9.54 to 6.90	0.753
Very likely	-5.67***	-8.80 to -2.54	< 0.001
Other categories	*Reference*		
			
*How concerned about meeting the legal and regulatory aspects of medicines*			
Very unconcerned	-9.20*	-16.83 to -1.56	0.018
Other categories	*Reference*		
			
N	192		

^*^ *p* < 0.05, ^**^ *p* < 0.01, ^***^ *p* < 0.001; other factors adjusted include age and gender.

As shown in Table 4, having a BSc or foundation degree is statistically significantly associated with a higher percentage of correct answers, relative to other routes, including Diploma of Higher Education and in-service training. Length of service had no statistically significant association. A confident respondent is more likely to show a higher outcome score. Conversely, the perceived knowledge score had a negative association with outcome, suggesting a potential mismatch between a respondent’s perceived and actual knowledge. Similarly, those respondents who claimed they need more training on average achieved a lower percentage score.

Those who are very likely to report *giving the wrong medicine with no harm caused to the patient* on average scored statistically significantly lower, compared to those who were less likely to report. Finally, those who claimed they were very unconcerned about *meeting the legal and regulatory aspects of medicines* were associated with a worse outcome.

## Discussion

The survey was completed by 251 out of approximately 1000 paramedics employed in the participating ambulance trust. Respondents scored a mean of 79% with a minimum of 52% and a maximum of 100% overall for correct answers on medications knowledge, the primary outcome. Noteworthy concepts scoring lower were route of administration, pain management, actions required for medication errors and incidents and recognition of an evidence-based tool. The knowledge score was statistically significantly associated with several characteristics and attitudes: perceived need for teaching, likelihood of reporting errors, perceived knowledge and likelihood of reporting errors, where higher self-rating in each of these was associated with a lower percentage correct knowledge score; and confidence rating, where a higher self-rating was associated with a higher percentage correct knowledge score. Having a foundation degree or a BSc was also associated with a higher correct knowledge score, compared to the scores of those who had completed in-service training or Diploma of Higher Education routes to becoming a paramedic. There is little evidence that length of service is associated with respondents’ knowledge.

The results demonstrate a range of correct knowledge on what are considered basic concepts surrounding medicines used in UK ambulance services. No literature pertaining to the same type of knowledge score among paramedics was found, but this study shows similarities with the online self-reporting error study of Vilke et al. (2007), in which 9.1% of paramedics reported committing a medication error within the last year, the actual rate being potentially higher due to non-reporting. The current results are apparently favourable compared with those reported by Boyle and Eastwood (2018), in which 20% of Australian paramedics enrolled on an intensive care course answered all 12 questions correctly. They are also favourable compared with those of Hoyle et al. (2020), who showed in an American study that 31% of all doses were incorrect after implementing a paediatric dosing reference system. The average self-reported knowledge score of 79% is slightly higher than the average score of 69% reported among registered Norwegian nurses in a cross-sectional MCQ (Simonsen et al., 2011), where 64% was considered the national level, lower scores being considered unsatisfactory.

It is perhaps reassuring to find that self-rated higher confidence was associated with increased correct knowledge, as was the perception of the need for more teaching with a lower correct knowledge score, as these might be indicative of a self-aware registered health care professional. Conversely, a higher self-rated perceived knowledge was associated with a reduced actual correct knowledge score. This is not entirely unexpected; for example, the self-assessed knowledge of pharmacy students has not been found to correlate with their actual knowledge (Naughton & Friesner, 2012); this appears to be a longstanding problem, with similar findings in college students in the 1970s (Galli, 1978).

A higher correct knowledge score being associated with those paramedics who had obtained their paramedic qualification via an initial route of higher education may suggest that the medicines-related elements within the education package are raising the knowledge surrounding medicines within paramedic practice in this trust. Lendraum et al. (2000) suggested that previous vocational training does not prepare ambulance personnel for their roles. Jones and Cookson (2000) support the move from protocol-directed care to credible education programmes.

The paramedic profession is changing rapidly. NHS England (2015a), in their consultation document on independent prescribing, recognise an advancing practice with an increasing skill set, and the necessity and advantages of treating in the community. All of these rely on promoting excellent, underpinning knowledge and professional standards to optimise safe care for patients (College of Paramedics, 2017). HCPC are changing their threshold entry level to becoming a paramedic (HCPC, n.d.) to BSc, meaning that all future registered paramedics will receive a higher education route to becoming a paramedic. However, in the Standards of Proficiency document from the HCPC (2014) there is little detail included about the different dimensions of the use of medicines, leaving a potential diversity in taught content. Differences of emphasis in higher education settings have been highlighted as impacting pharmacy knowledge for pharmacy and medical students (Keijsers et al., 2014).

The results suggest that while addressing medicines knowledge topics is important, taking account of perceptions of knowledge and confidence is also necessary. Those participants who did not score 100% in the legal questions may find their actions placing them or their trust in a position of working otherwise than in accordance with legislation.

In moving from protocol-based systems to more advanced systems of medicines administration, the possibility exists that there is a need for an increase in the knowledge and respect surrounding medicines for patients to receive the best and safest care, and for individuals and trusts to function within the law and regulations. Education is clearly one way to address the deficit and is the main focus of this study, but this merely improves the functioning at the ground level and, especially in the cases of legal and regulatory issues, would require a more integrated approach at both the ground and senior levels. The CQC in their report identified the need to embed safety procedures pertaining to medicines into everyday practice that can be sustained by front line ambulance staff and managers (CQC, 2017b). It is that compatibility and integration of the levels that achieves a higher professionalisation, as highlighted by the findings of an ethnographic study in a UK ambulance service from 2012 (McCann et al., 2013). In developing an integrated improvement strategy for medicines optimisation (NICE, 2015), it is likely that the particularities of an emergency ambulance service’s paramedics’ lone working are taken into consideration. The Health and Safety Executive published a set of guidance notes on the importance of training where there is limited supervision and, while not specific to medicines, it highlights employers’ responsibilities to ensure staff are competent, trained and able to recognise when they should get advice. It states that supervision should be provided based on assessment of risk (HSE, n.d.). Such an approach is likely to require leadership, with individual paramedic leaders considered to need to balance the constituent parts of the leader, the follower, common goals and the situation (Johnson et al., 2018), the situation here being the optimisation of paramedic knowledge within a system for medicines management.

### Limitations

This study acknowledges a number of limitations. Respondents might have had multiple attempts, since IP addresses were not monitored. There was no time cut off, so respondents could have looked up the answers or asked someone. There is also a risk of social desirability bias in responses. The study was limited to one UK ambulance trust and the sample, although adequate for the analyses performed, was relatively small. Therefore, there may be lack of power for Little’s MCAR test; this test is based on data from respondents, so the most challenging problem related to missing data is the low response rate, and it is uncertain if the sample is representative. The final regression results were based on a sample of 192, approximately 20% of the potential population, so it is important to note that the generalisation of this result may be limited. It is thus important that future validation studies be conducted to verify the findings detailed in this article. This study’s results will inform a sample size calculation. The focus of this survey was also on the paramedic as an individual, and it was not able to address the system-level contributions to medicines safety; further research is required.

## Conclusion

This single-site UK-based survey of medicines knowledge and attitudes in paramedics has highlighted variation in knowledge among respondents, with some incorrect knowledge in the majority. A higher correct knowledge was associated with higher self-rated confidence, lower self-reported knowledge and being very likely to report any errors, as well as with a higher education-based initial route into the paramedic profession. Although the respondents only totalled an estimated quarter of the population and cannot be presumed to be a representative sample, the results indicate a need to focus on medicines-specific further education for paramedics – particularly those who have not experienced longer formal education integrating a focus on confidence and self-perceived knowledge – and to enhance and embed integrated improvement strategies. Further research is required with larger, multi-site samples, and to evaluate the impact of education packages developed in the context of other potential components of a package of improvement for safe medicines optimisation.

## Acknowledgements

We would like to acknowledge the contribution and support of the staff from the trust R&D who approved the study, as well as the ambulance service paramedic respondents.

## Author contributions

SL was chief investigator for this study, having conceived the idea and designed the study and its materials in collaboration with MH and CW. SL collected the data, which was analysed and interpreted by SL, CW (who conducted the statistical analyses) and MH. SL prepared the draft manuscript, supported by CW and MH, who provided academic research oversight throughout the study. All authors critically reviewed and approved the final manuscript. SL acts as the guarantor for this article.

## Conflict of interest

None declared.

## Ethics

Favourable ethical opinion was gained from the Faculty Research Ethics Committee of the joint Faculty of Health, Social Care and Education at Kingston University and St George’s, University of London (ref number 2018-11-003).

## Funding

Funded by a grant from the College of Paramedics.

## Appendix: The survey invitation

Version number 2

Date 10/12/18

IRAS ID 253917

A scoping project on the knowledge, attitudes and practices of Paramedics regarding pharmacology and the legal and regulatory issues of medicine management and administration within the UK NHS ambulance service.

We would like to invite you to complete a questionnaire to gain a better understanding of how Paramedics in your trust utilise medicines. We are interested in what you know and how you feel about using medicines. The results will inform the content of the teaching on medicines that your trust is planning. The questionnaire is anonymous and there is a prize draw to win a £25 book voucher. We expect it to take less than 20 minutes to complete. You must be a registered Paramedic to complete the questionnaire.

The link to the survey is https://kingston.onlinesurveys.ac.uk/a-scoping-project-on-the-knowledge-attitudes-and-practice.

For more information, or a paper copy of the survey, please contact Samantha Laws in the Department of Paramedics, Kingston University and St George’s University of London at s.laws@sgul.kingston.ac.uk.
